# A review of antibody, aptamer, and nanomaterials synergistic systems for an amplified electrochemical signal

**DOI:** 10.3389/fbioe.2024.1361469

**Published:** 2024-03-13

**Authors:** Resmond L. Reaño, Erwin C. Escobar

**Affiliations:** Department of Engineering Science, College of Engineering and Agro-industrial Technology, University of the Philippines Los Baños, Los Baños, Philippines

**Keywords:** dual system approach, sandwich format, monoclonal antibody, electrochemical aptasensor, nanocarrier

## Abstract

The synergy between biomolecules with inorganic nanomaterials and nanoparticles has been investigated over the past years, primarily to improve biomarker reception, generate signals, and amplify the signals generated. In this paper, several articles on aptamer-based and antibody-based electrochemical biosensors that target antigens were examined. Among the key characteristics identified were the electrochemical platform development, which includes the usage of nanomaterials as electroactive or electrocatalytic labels, crosslinking of the biological agent with inorganic compounds, and electrode coating to provide an electronic source and support efficient electron transfer. A single approach using labeled or unlabeled biological receptors has become advantageous due to its simple architecture and more straightforward application method. However, the dual system approach allows the incorporation of more nanomaterials to boost the signal and add more features to the electrochemical system. The dual system approach uses a capture and reporter probe in a competitive or sandwich detection format. The reporter probe is often labeled by an electroactive or electrocatalytic compound or immobilized in a nanocarrier, resulting in an increase in measured peak current in proportion to the target’s concentration. The reported limit of detection and linear range for each platform is presented to assess its efficiency. Generally, the dual system aptasensor showed higher sensitivity, stability, and reproducibility than the immunosensor in comparable settings. The aptasensor showed promising results for the development of point-of-care type applications.

## 1 Introduction

Biosensor offers advantages such as rapid, more straightforward sample processing and implementation and cost-effective, sensitive, and stable detection method (Wei et al., 2018). It can be utilized in various fields, such as medicine, the food and packaging industry, agriculture, and environmental monitoring (Jafari et al., 2019). The biosensor’s analytical sensitivity and selectivity rely heavily on a stable, strong, and specific binding between the molecular recognition element—the bioreceptor, and the target biomarker (Kondzior and Grabowska, 2020). Antibodies have become a popular candidate for biosensor development owing to their high affinity and specificity to their target biomolecule. Antibody-based Enzyme-Linked Immuno-Sorbent Assay (ELISA), the gold standard for all immunoassays is still popular and is used worldwide in different fields of application, particularly in clinical diagnostics. With the advancements in analytical and bioanalytical chemistry, the incorporation of antibodies directly to the signal transducer’s surface gave birth to a combined immunoassay and biosensor technology termed immunosensor (Jafari et al., 2019; Popov et al., 2021).

An immunosensor is a biosensor that uses antibody (Ab), either polyclonal (pAb) or monoclonal (mAb), as a capture and signaling element. Such antibody forms a stable immunocomplex with the antigen (Ag), generating a measurable signal. In contrast with an immunosensor, in an immunoassay, the signal recognition takes place elsewhere (Mollarasouli et al., 2019; Shen et al., 2020). Among the limitations of using antibodies are difficulty in chemical modification, high cost of production, and low stability at high temperatures (Wei et al., 2018). Aptamer-based electrochemical biosensors were developed to overcome these limitations.

Aptamers gained research interest since it was revealed in 1990 as a potential rival to antibodies in terms of its diverse application due to its ability to form 2D and 3D shapes that help them to recognize and bind to their cognate target with high affinity and specificity (Jayasena, 1999). Aptamers are short single-stranded nucleic acids (can be DNA or RNA) that are selected from a set of random DNA or RNA library and synthesized *in vitro* using a method called Systematic Evolution of Ligands by Exponential Enrichment (SELEX) (Sypabekova et al., 2017). Aptamers are stable in complex environments and highly resistant to denaturation and degradation when modified and optimized appropriately. A biosensor that uses an aptamer as a molecular recognition element or bioreceptor is called an aptasensor (Bezerra et al., 2019; Anand et al., 2021; Bhardwaj and Kumar Sharma, 2022; McKeague et al., 2022).

Several transduction techniques can be used for biosensor development, which includes optical, chemiluminescent, electro-chemiluminescent, colorimetric, fluorometric, piezoelectric, and electrochemical. Most of these techniques are complex, time-consuming, and require sample pre-treatment and personnel training to perform the procedure. Electrochemical techniques received much attention due to their high sensitivity and selectivity, simple design, and rapid detection without requiring expensive and complex equipment. Electrochemical techniques are easily integrated into the biosensor, and the resulting device can be miniaturized, making the electrochemical biosensor highly applicable for point-of-care testing (Marques et al., 2014; Zhong et al., 2020). Electrochemical biosensors can be operated using low-voltage disposable batteries. It can also obtain its power source from other electronic devices such as cell phones, tablets, laptops, and computers when accompanied by a computer application. Electrochemical biosensors can detect multiple analytes simultaneously (multiplexing) (Arkan et al., 2015). In such biosensors, antibodies and aptamers can be utilized as bioreceptors, often immobilized on the electrode surface using appropriate chemistry. Stable Ab-Ag or Ap-Ag complex formation generates electrical signals, such as changes in electrode potential, current, or capacitance (Shen et al., 2020). Furthermore, aptamers offer an additional advantage over antibodies as, unlike the latter, aptamers can undergo a target-induced structural change. When labeled with a redox molecule, this structural change can be quantified proportionately to the analyte concentration (Das et al., 2019).

The key challenges to improving the electrochemical biosensor’s performance include signal amplification and electrode stability. The combination of biological and inorganic nanomaterials has been explored, including labeling of bioreceptors, using various bio-linking techniques, and incorporating electronic sources. A dual system has become a popular technique that allows the incorporation of more nanomaterials into the platform, resulting in a more flexible biosensor application.

This review aimed to obtain insight into the answers to the following research questions: 1) What strategies are used to enhance the electrochemical signal and lower the limit of detection? 2) What techniques are used to incorporate the biological and inorganic nanomaterials into the electrode assembly? 3) What is the impact of using a dual system approach in electrochemical signal amplification? The reviewed articles in this paper encompass single systems with unlabeled bioreceptors to more complex dual systems decorated with various electronic nanomaterials.

In this review paper, a biosensor refers to an electrochemical immunosensor or aptasensor, while a bioreceptor pertains to either an antibody or an aptamer. This review paper focuses on the recent application of aptamer and antibody as bioreceptors in a single system and dual systems composed of aptamer-aptamer (or complementary DNA), antibody-secondary antibody, and antibody-aptamer in developing electrochemical biosensors. Articles on electrochemical biosensor development against various protein biomarkers reported from 2012 to 2022 were considered, thoroughly studied, analyzed, and presented in this review.

## 2 Electrochemical signal amplification strategies

Typically, an electrochemical biosensor comprises an electrode with an interface architecture where the biological event occurs. This interaction includes the specific binding of the analyte to the bioreceptor, producing an electrochemical signal. In the transducer, this signal is detected and converted to an electronic signal and is sent to a computer for processing. Computer software converts the electronic signal into a meaningful physical quantity presented to the human operator through an interface. This technique’s advantages are simplicity, rapidity, cost-effectiveness, and high sensitivity. Electrochemical biosensors are easy to miniaturize, are independent of sample turbidity, and are compatible with novel microfabrication techniques. Since an electronic signal is produced directly after an electrochemical reaction occurs, expensive signal transduction equipment is not required (Hayat and Marty, 2014).

Due to the advent of screen-printing technology, miniaturized electrodes have become more feasible. Carbon paste electrodes are prepared using paraffin or mineral oil, which can be printed on a screen. Nanoparticles can also be incorporated into the mixture while preparing the paste electrode. Recently, paper-based biosensors have become a viable choice for electrode fabrication since they are readily available, inexpensive, disposable, and biocompatible. Due to its simple fabrication method, it is a strong candidate for point-of-care (POC) applications. The paper electrode has been used to develop electrochemical immunosensors (Fan et al., 2019) and aptasensors (Wei et al., 2018). The techniques used include wax printing, plasma treatment, UV photolithography, screen printing, and laser treatment (Fan et al., 2019).

The change in electrochemical signal from the protein-protein interaction on the electrode’s surface is usually low and often requires a signal amplification method. Nanomaterials were used either 1) to modify the electrode’s surface by coating or by labeling the immobilized bioreceptor and 2) to introduce the electroactive or electrocatalytic nanomaterials into the system as a secondary probe (Johari-Ahar et al., 2015).

### 2.1 Electrochemical techniques

Electrochemical techniques measure the response of an electrochemical cell containing an electrolyte upon the application of electric current by the conductive electrodes immersed in that electrolyte (Doménech-Carbó et al., 2015). The applied electric current results in the loss (oxidation) or gain (reduction) of electrons of a given material in the electrolyte or embedded into the electrode. These redox reactions provide information such as concentration, kinetics, reaction mechanism, and other behaviors of a species in a solution (Naresh and Lee, 2021). This information, obtained as an electrochemical signal, is translated into meaningful values, which are used to evaluate the performance of an electrochemical biosensor.

Electrochemical techniques can be classified as amperometry, potentiometry, and coulometry. Amperometry measures the current in response to applying a constant or pulsed potential (Costa et al., 2022). Voltammetry, a subclass of amperometry, is the most applied technique in diagnostics and environmental analysis, particularly cyclic voltammetry (CV), differential pulse voltammetry (DPV), and square wave voltammetry (SWV), because of its simplicity and speed (Magar et al., 2021). Voltammetry consists of the records of current measured using a working electrode as a function of the potential difference between the working electrode and reference electrode. Typically, a third electrode, the counter electrode, minimizes the current passing through the working electrode (Doménech-Carbó et al., 2015).

In a reversible system, more intense signals are obtained using SWV, increasing the sensitivity compared to other voltammetric techniques. SWV is more rapid and sensitive than DPV due to the absence of interference caused by the background current (Costa et al., 2022).

Potentiometry is based on the Nernst equation, which relates the potential produced by the galvanic cell to the concentration of the electroactive species. However, this is only valid under equilibrium or thermodynamic conditions. (Westbroek, 2005).

Coulometry measures the total charge or the number of coulombs spent as an analyte is exhaustively converted from one oxidation state to another at the working electrode. This is an absolute process wherein the current passed is measured to calculate the number of electrons passed (Houssin et al., 2021). Coulometry was an analytical technique popular in the twentieth century and is now finding applications in miniaturized systems.

An emerging electroanalytical method in biosensor applications is electrochemical impedance spectroscopy (EIS), which is used for characterizing electrodes and performing impedimetric analysis (Doménech-Carbó et al., 2015). In EIS, the sinusoidal response (current or voltage) is monitored as an equilibrium or steady electrochemical system undergoes perturbation via the application of a sinusoidal signal (AC voltage or AC current, respectively) at a varied range of frequencies (Lazanas and Prodromidis, 2023).

In an electrochemical immunosensor, EIS and SWV are the most popular techniques used due to their high sensitivity, with the limit of detection obtained at the picomolar level and over a wide dynamic range. SWV was determined to be rapid, efficient, cost-effective, and inexpensive when applied to label-free electrochemical immunosensors (Liu et al., 2010).

Aside from the electrochemical techniques used, the electrode type contributes to the sensitivity and selectivity of an electrochemical biosensor. Electrodes are usually based on carbon and noble metals, with carbon-based electrodes being more prevalent in biological research due to their high sensitivity and other benefits. Noble metals offer advantages in developing inexpensive multiplexed electrochemical sensors (Zachek et al., 2008). Carbon and gold-based electrodes have become popular due to their high conductivity, biocompatibility, and stability, which are crucial for biosensor development. Recent studies include hybrid electrode systems that take advantage of the benefits of various materials.

### 2.2 Electrode’s surface modification with nanomaterials

The electrode’s surface is modified with nanomaterials to increase the surface area and create a more favorable environment, leading to excellent biocompatibility, higher conductivity, and stability (Wei et al., 2018). Selecting appropriate nanomaterials is critical to improving the performance of an electrochemical biosensor (Jafari et al., 2019). Recent research focuses on modifying the electrode’s surface with nanomaterials to promote electron transfer, signal amplification, and improvement of low-end detection limit (Freitas et al., 2019). Several studies showed that signal amplification can be achieved using nanomaterials such as graphene (G), quantum dots (QD), and metal nanoparticles (MNP) (Valipour and Roushani, 2017). Gold nanoparticles (AuNP) are often used to coat the electrode of the aptasensor due to the ease of immobilizing thiolated aptamers (Popov et al., 2021).

AuNP is a metallic nanoparticle with a high specific surface area, good biocompatibility, and high surface-free energy. It can also bond with the amino functional group (−NH_2_) and thiol group (−SH) (Wei et al., 2018). AuNP can be used either as the electrode or integrated into carbon-based electrodes. It is often prepared by citrate reduction of chloroauric acid in aqueous solution using sodium citrate (Arkan et al., 2015). AuNP is used to immobilize biological substances due to its physicochemical properties. Thiolated biomolecules are quickly immobilized on AuNP’s surface. Reports showed that protein immobilization on AuNP helps preserve the activity of the biomolecule. AuNP can help maintain the immunoactivity of the antibodies. Aside from that, AuNPs are highly conductive, facilitating direct electron transfer between redox species and bulk electrode materials, which is ideal for electrochemical sensing (Gasparotto et al., 2017).

Silver, platinum, and palladium nanoparticles can also be used to improve the sensitivity and performance of electrochemical biosensors. The advantages of these nanoparticles include strong adsorption ability, simplicity of the preparation process, cost-effective manufacturing process, high conductivity, and large specific surface area (Valipour and Roushani, 2017).

Carbon-based nanomaterials are used in various applications, such as detecting biomolecules, proteins, and nucleic acids (Raouafi et al., 2019). Graphene is one of the most promising carbon-based nanomaterials in designing electrochemical biosensors (Wei et al., 2018; Karaca and Acaralı, 2023). Graphene oxide (GO) has unique characteristics such as large surface area, good water dispersibility, facile surface modification, and photoluminescence (Zhu et al., 2015). GO can readily adsorb aptamers on its surface and is often combined with MNP for firmer protein immobilization. Carboxylic acid functionalized GO can be used to immobilize aminated aptamer via covalent bonding (Johari-Ahar et al., 2015).

Quantum dots are semiconductors used successfully to amplify faint sensing signals. QD capped with organic linkers can boost the signal generated from electrochemical measurements (Johari-Ahar et al., 2015). Among the novel nanomaterials are graphene quantum dots (GQD), which are inexpensive, have high aqueous dispersibility, are ultra-small, and can be modified with a functional group. GQD exhibits a redox behavior by applying electrochemical techniques (Srivastava et al., 2018; Jafari et al., 2019; Kansara et al., 2022). GQD is superior to other semiconductor QDs in terms of low cytotoxicity, biocompatibility, ease of production, chemical inertness, and resistance to photobleaching. GQD can be thiolated to promote the immobilization of metal nanoparticles. Thiolated GQD also forms stable colloidal suspensions in various solvents, including ethanol and dimethylformamide. The water solubility of thiolated GQD is also lower than that of oxidized GQD (Valipour and Roushani, 2017). Chitosan (CH) is used to avoid restacking of GQD and provides a stable film or matrix for immobilizing the biomolecule (Srivastava et al., 2018). Carbon quantum dots or carbon dots (CD), <10 nm fluorescent nanoparticles, are a new addition to the carbon nanomaterial family. GQD and CD have been commonly used for biosensors, bioimaging, and targeted drug delivery research for cancer theranostics (Jana and Dev, 2022).

Nanozymes are nanomaterials with properties like enzymes and are known to have advantages such as low production cost, ease of mass production, and robustness. Iron oxide nanoparticles (IONP), other metal oxides, and metal-organic framework (MOF) have been discovered to possess intrinsic enzyme-like activity similar to horseradish peroxidase (HRP) (Sun et al., 2019). A MOF is a porous crystalline material that has gained popularity due to its stable and tunable pore sizes and high surface area. MOF inherits the advantages of its parent material, thus significantly boosting its application. Nanohybrid electrocatalysis can be performed by combining MOF and bimetallic nanoparticles carrying the bioreceptors (Sun et al., 2019). Covalent organic framework (COF) belongs to the highly porous materials synthesized for water treatment, energy, gas storage, and biosensing applications (Altaf et al., 2021).

Zhong et al. (2020) used ferrocene nanoparticles, an organometallic compound, as the electrochemical signal indicator that passes through the carbon nanotube, acting as the conductive layer. This study used a surface-confined setup with a label-free protein as the receptor. This study demonstrates the use of nanoparticles to resolve the problem with low sensitivity without labeling the protein while also providing a reagent-less approach.

Functionalized metal nanoparticles such as iron oxide (Fe_3_O_4_) have been used in biosensing systems due to their biocompatibility, signal amplification, and ability to form covalent bonds with antibodies via their functional group (Emami et al., 2014). Magnetic IONP was used to immobilize antibodies efficiently to detect human epidermal growth factor receptor 2 (HER2) (Emami et al., 2014; Shamsipur et al., 2018). Polyethylene glycol (PEG) has been used as an antibody linker to MNP. PEG provides enough space to allow more antibodies to bind with the MNP, thus creating a more effective combination with the target (Emami et al., 2014). MNP is easy to collect, wash, and handle using magnets or magnetic bars. IONP has been utilized as a magnetic core of a bio-conjugated nanoparticle and is often coated with trimethoxy-silane compounds for biomolecule conjugation (Marques et al., 2014). Zinc oxide (ZnO) has been utilized in biosensor platforms due to its high isoelectric point (IEP∼9.5). Due to its semiconductor properties, ZnO provides an effective channel for electron transport during redox. AuNP can be synthesized directly into ZnO and Zn-based MOF matrix (Gasparotto et al., 2017).

### 2.3 Labeling and detection format

Three types of detection formats are commonly used to facilitate biomarker detection. The simplest is the direct type, wherein the biomarker attaches to the bioreceptor immobilized on the electrode’s surface. This method is more straightforward, easy to implement, quick, and desirable for miniaturized sensors (Emami et al., 2014).

**FIGURE 1:**
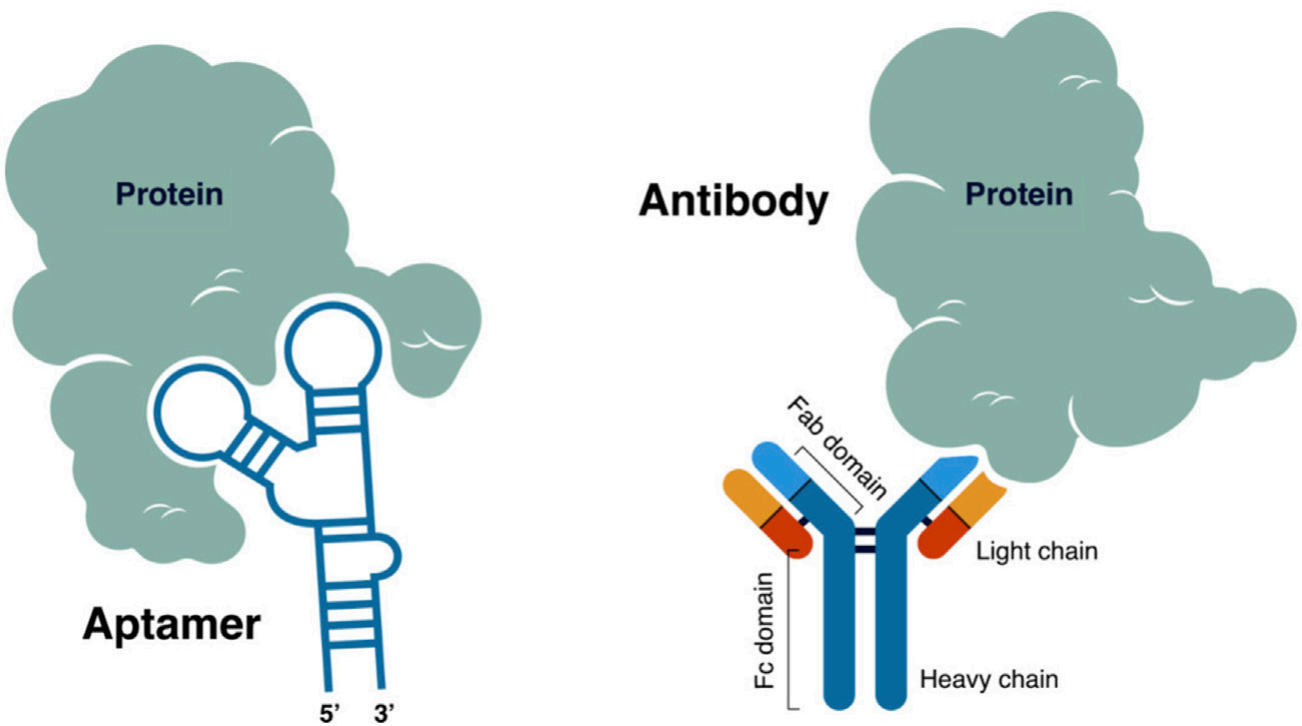
Label-free approach—the interaction between the biomarker and the biological receptor decreases the electrochemical signal.

**FIGURE 2:**
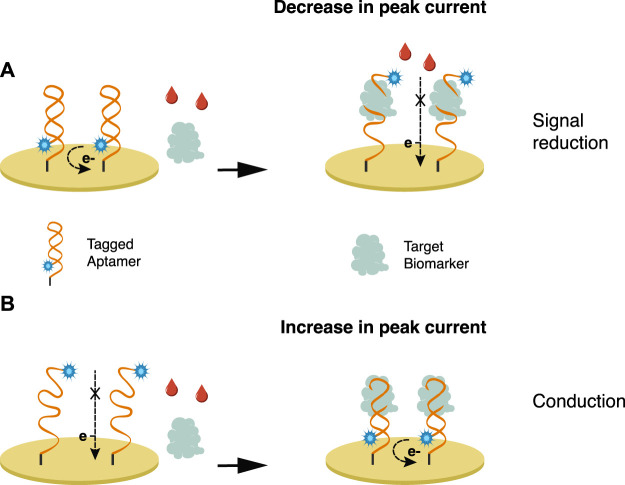
Bioreceptors are tagged with an electroactive label wherein the interaction with the biomarker either promotes **(A)** signal reduction or **(B)** conduction.

In a label-free approach (Figure 1), the attachment of the biomarker to the electrode’s surface hinders the electron transfer and decreases signal intensity. The probe can be labeled (Figure 2) to produce an electrochemical signal, promoting either signal conduction (signal-on) or reduction (signal-off) (Zhong et al., 2020). A redox indicator, such as methylene blue and ferrocene/ferrocyanide redox couple—which is sensitive to the protein charge and surface blocking, can be used (Salimian et al., 2017; Zhong et al., 2020).

Immunolabeling refers to labeling antibodies or antigens to catalyze complex formation and improve biosensor sensitivity (Shen et al., 2020). Two types of labels are commonly used: 1) an electroactive label or 2) an electrocatalytic label, such as an enzyme that catalyzes the production of an electroactive product (Kondzior and Grabowska, 2020). Standard labels comprise nanomaterials or nanoparticles, enzymes, radioisotopes, luciferin, and electronic dense substances (Shen et al., 2020). Among the most valuable labels are enzymes, such as horseradish peroxidase (HRP), glucose oxidase (GOD), alkaline phosphatase (ALP), or electroactive molecules such as ferrocene, ferrocyanide, methylene blue (MB), platinum and cadmium quantum dots (QDs), and other nanoparticles. Table 1 shows that antibodies are often labeled with horseradish peroxidase (HRP) or alkaline phosphatase (AP), assuming the exact mechanism as the commonly known ELISA technique.

**TABLE 1:** Labeled and dual system approaches to lowering the limit of detection (LOD).

Aptamer/antibody and nanomaterial synergy	Electrochemical indicator or reporter probe	Electrochemical technique	Target biomarker	Sample	Performance	References
Single-labeled or unlabeled aptamer or antibody						
Aptamer-SH/AuNP/SPCE	None	DPV	PSA	Clinical human serum	LOD: 0.077 pg/mL	Hassani et al. (2020)
					LR: 0.001–200 ng/mL	
Aptamer-SH/AuNP/THI^a^/rGO/SPCE	None	DPV	PSA	Clinical human serum	LOD: 10 pg/mL	Wei et al. (2018)
					LR: 0.05–200 ng/mL	
Aptamer-NH_2_/GQD-CoPc^b^/GCE	None	EIS, DPV	PSA	PSA solution in PBS and with BSA, glucose, and L-cysteine	LOD: 0.018 ng/mL	Nxele and Nyokong (2021)
					LR: 0.034–0.057 ng/mL	
Aptamer-NH_2_/CH-GQD@AuNR/SPCE	None	CV, DPV, EIS	PSA	Spiked human serum	For CV LOD: 0.14 ng/mL Sensitivity: 3.7 μA ng/mL	Srivastava et al. (2018)
					For DPV LOD: 0.14 ng/mL Sensitivity: 2.5 μA ng/mL	
					For EIS LOD: 0.14 ng/mL Sensitivity: 35 kΩ ng/mL	
mAb/CH-GQD@AuNR/SPCE	None				For CV LOD: 0.14 ng/mL Sensitivity: 4.6 μA ng/mL	
					For DPV LOD: 0.14 ng/mL Sensitivity: 2.39 μA ng/mL	
					For EIS	
					LOD: 0.14 ng/mL	
					Sensitivity: 25.6 kΩ ng/mL	
Aptamer-NH_2_/MPA^c^/AuNP@Gold E	None	EIS	HER2	PBS solution	LOD: 5 ng/mL	Chun et al. (2013)
					LR: 10^–5^–10^2^ ng/mL	
Aptamer-NH_2_/SNGQD^d^@AuNP/GCE	None	EIS	HER2	Spiked human serum (1:500 dilution)	LOD: 0.0489 ng/mL	Centane and Nyokong (2022)
Aptamer-NH_2_/CoP-BNF/GCE					LOD: 0.0259 ng/mL	
Aptamer/SNGQD@AuNP/CoP-BNF^e^/GCE					LOD: 0.0112 ng/mL	
Ab/SNGQD@AuNP/GCE					LOD: 0.1072 ng/mL	
Ab/CoP-BNF/GCE					LOD: 0.0454 ng/mL	
Ab/SNGQD@AuNP/CoP-BNF/GCE					LOD: 0.0327 ng/mL	
Ab/rGO-Au/GCE	None	SWV, EIS	PSA	Spiked human serum samples	SWV	Assari et al. (2019)
					LOD: 2 pg/mL	
					LR: 25–55 fg/mL and 1–36 ng/mL	
					EIS	
					LOD: 60 pg/mL	
					LR: 1.8 pg/mL –41 ng/mL	
Ab/AuNP@ZnO nanorod/SPGE	None	CV	CA125	PBS as support electrolyte	LOD: 2.5 ng/μL	Gasparotto et al. (2017)
Ab/Streptavidin/MOF-808@CNT/GCE	None	DPV	CA125	Tested on patient serum samples	LOD: 0.5 pg/mL	Biswas et al. (2021)
					LR: 0.001–0.1 and 0.1–30 ng/mL	
Ab/AuNP/THI/rGO/SPCE	None	DPV	CA125	Quality control serum samples	LOD: 0.01 U/mL	Fan et al. (2019)
					LR: 0.1 U/mL to 200 U/mL	
Ab/AgNP@GQD/GCE	None	DPV	CA125	Riboflavin solution	LLOQ: 0.01 U/mL	Jafari et al. (2019)
					LR: 0.01–400 U/mL	
Ab/MPA-AuNP@SiO_2_-CdSe QD/Gold E	K_3_Fe(CN)_6_ is used as an indicator	CV, EIS	CA125	Spiked human serum	LOD: 0.0016 U/mL	Johari-Ahar et al. (2015)
					LR: 0–0.1 U/mL	
Ab/R1^f^/SPGE	A quinone-based compound in R1	DPV	CEA	Spiked human serum sample	LOD: 0.33 ng/mL	Pavithra et al. (2018)
					LR: 1.0–100 ng/mL	
Aptamer/PEG^g^-Gold E	MB and K_3_Fe(CN)_6_ is used as indicator	CV	HER2	Buffer and 1% human serum	LOD: 1 pM	Salimian et al. (2017)
					LR: 1 pM–10 nM	
Ab/AuNP/HDT^h^/AuNP@MW-CILE^i^	K_3_Fe(CN)_6_ is used as an indicator	EIS	HER2	Human serum	LOD: 7.4 ng/mL	Arkan et al. (2015)
					LR: 10–100 ng/mL	
Ab/Fc-PEI/SWNT/ITO^j^	Fc is used as an indicator attached to ITO	DPV	HER2	Human serum (diluted 20 times)	LOD: 0.220 ng/mL	Zhong et al. (2020)
					LR: 1.0–200 ng/mL	
Ab-bioconjugate^k^/Cys/MPA/AuNP/Gold E	IONP core	CV, DPV, EIS	HER2	Human serum samples	LOD: 0.995 pg/mL	Emami et al. (2014)
					Sensitivity: 5.921 μA mL/ng	
					LR: 0.01–10 ng/mL and 10–100 ng/mL	
MB-Aptamer/PLLF^l^/SPCE	MB from labeled 1° aptamer	DPV	HER2	Human serum (after albumin depletion)	LOD: 3 ng/mL	Bezerra et al. (2019)
					LR: 10–60 ng/mL	
MB-Aptamer-SH/Gold E	MB from labeled 1° aptamer	DPV	PSA	Human samples	LOD: 50 pg/mL (0.050 ng/mL)	Sattarahmady et al. (2017)
					LR: 0.125–128 ng/mL	
Dual Antibody–Secondary antibody system						
pAb_1_/SPCE(passive Ab immobilization)	pAb_2_-Biotin/Streptavidin-HRP	CV	HER2	Tested on human serum samples	LOD: 4 ng/mL	Tallapragada et al. (2017)
	TMB is used as a substrate (*sandwich format*)				LOQ: 5 ng/mL	
					LR: 5–20 and 20–200 ng/mL	
mAb_1_/SPCE	mAb_2_-Biotin/S-AP^m^	LSV	HER2	Human serum samples	LOD: 4.4 ng/mL	Marques et al. (2014)
	3-indoxyl phosphate and silver ions were used as substrate (sandwich format)					

Ab_1_/AuNP/MWCNT-SPCE	Ab_2_-Biotin/S-AP	LSV	HER2-ECD	Human spiked serum	LOD: 0.16 ng/mL	Freitas et al. (2019)
Ab_1_/SPCE*	3-indoxyl phosphate and silver ions were used as substrate (*sandwich format*)				LOD*:8.5 ng/mL	
Nb/SPCE (EDC/NHS coupling)	HRP-Nb	CV	HER2	Cell lysate spiked with HER2	LOD: 1 μg/mL	Patris et al. (2014)
	(H_2_O_2_ and hydroquinone were used as substrate) (*sandwich format*)				LOQ: 4.4 μg/mL	
pAb/IONP@MWCNT-COOH/GCE	HRP-mAb	DPV	PSA	Human serum samples	LOD: 0.39 pg/mL	Shamsazar et al. (2021)
	H_2_O_2_ → H_2_O				LR: 2.5 pg/mL–100 ng/mL	
	HRP_(red)_ → HRP_(ox)_ (*sandwich format*)					
Biotin-mAb/streptavidin/ABA^n^/nano-TiO_2_-CPE	THI/HRP-pAb (*sandwich format*)	CV	PSA	PSA solutions	LOD: 200 pg/mL	Biniaz et al. (2017)
				Human serum samples	LR: 0.10–5.0 ng/mL and 5.0–100 ng/mL	
Ab_1_/CA15-3/rGO-NH_2_/SPE	HRP-Ab_2_	DPV	Anti-CA15-3	Human serum samples	LOD: 0.0001 ng/mL	Patil et al. (2022)
Dual aptamer system						
MB-Aptamer-NH_2_/GO-CO_2_H/SPCE (MB is intercalated)	cDNA-NH_2_ (*competitive format*)	CV, DPV	PSA	PBS Solution	LOD: 0.064 pg/mL	Raouafi et al. (2019)
				Validated in spiked human blood serum	LR: 1 pg/mL–100 ng/mL	
Aptamer_1_-SH/GNF^o^@SPCE	Aptamer-probe A duplex	DPV	CA125	Spiked biological samples	LOD: 5.0 pg/mL	Chen et al. (2019)
Aptamer_1_–hairpin-like structure	MB-Aptamer_2_ in solution				LR: 0.05–50 ng/mL	
	Target binds to duplex, releasing probe A. Probe A opens Aptamer_1_, MB-Aptamer_2_ attaches to Aptamer_1_.(*indirect format*)					
Aptamer-NH/TTCA^p^/AuNP@SPCE	Hydrazine-Phosphate-Aptamer (*sandwich format*)	CA	cTnI	Human serum (male AB plasma)	LOD: 24 pg/mL	Jo et al. (2017)
					DR: 0.024–2.4 ng/mL	
Aptamer/NTH/SPGE	(NP1) Aptamer/Cu@Au/Fe_3_O_4_@UiO	DPV	cTnI	Human serum sample	LOD: 16 pg/mL	Sun et al. (2019)
	(NP2) cDNA/Au@Cu (*sandwich format*)				LR: 0.05–100 ng/mL	
Aptamer_1-2_-NH/MPA-AuNP/3DGH^q^-GCE (*duplex system*)	Aptamer_3_/AuNP/HGN^r^	DPV	CEA	Clinical serum samples	LOD: 11.2 pg/mL	Shekari et al. (2021)
	Aptamer_4_/AuNP/Fc/Graphene		CA15-3		LOD: 0.112 U/mL	
Dual antibody-aptamer system						
mAb/poly-DPB^s^(AuNP)/GCE	Hyd-Aptamer-SH/AuNP	CV, SWV	HER2	25-fold diluted human serum	LOD: 0.037 pg/mL	Zhu et al. (2013)
					LR: 0.1 pg/mL–10 ng/mL	
Ab/Gold E	Aptamer-Au-Cysteamine conjugate	EIS, CV, and DPV	Tau-381	Human serum	LOD: 0.42 pM	Shui et al. (2018)
					LR: 0.5–100 pM	
Aptamer-Biotin/Streptavidin-MB	Ab/AuNP	ASDPV^t^	EGFR^u^	Human serum	LOD: 50 ng/mL	Ilkhani et al. (2015)
					LR: 1–40 ng/mL	

^a^ THI–Thionine.

^b^ GQD-CoPc–Graphene quantum dots–Co phthalocyanine.

^c^ MPA–Mercaptopropionic acid.

^d^ SNGQD–Sulfur-nitrogen doped graphene quantum dots.

^e^ CoP-BNF–Cobalt porphyrin binuclear framework.

^f^ R1–lawsone + 2-mercaptoethylamine.

^g^ PEG–polyethylene glycol.

^h^ HDT–1,6-hexanedithiol.

^i^ MW-CILE–multi-walled carbon nanotube–ionic liquid electrode in PVC tube.

^j^ Fc-PEI, Ferrocene in polyethylene imine; SWNT, single walled carbon nanotube; ITO, indium-tin oxide electrode.

^k^ Ab-bioconjugate–IONP/3-amino-propyltrimethoxysilane (APTMS)/PEG/thiol-antibody.

^l^ PLLF–poly-L-Lysine film.

^m^ S-AP–streptavidin-alkaline phosphatase.

^n^ ABA–4-amino benzoic acid, CPE–carbon paste electrode.

^o^ GNF–gold nanoflower.

^p^ TTCA–5,2’:5′2″-terthiophene-3′-carboxylic acid.

^q^ 3DGH–three-dimensional graphene hydrogel.

^r^ HGN, Hemin-graphene hybrid nanosheets.

^s^ DPB–2,5-bis(2-thienyl)-1H-pyrrole-1-(p-benzoic acid).

^t^ ASDPV, Anodic stripping differential pulse voltammetry.

^u^ EGFR, Epidermal growth factor receptor.

**FIGURE 3:**
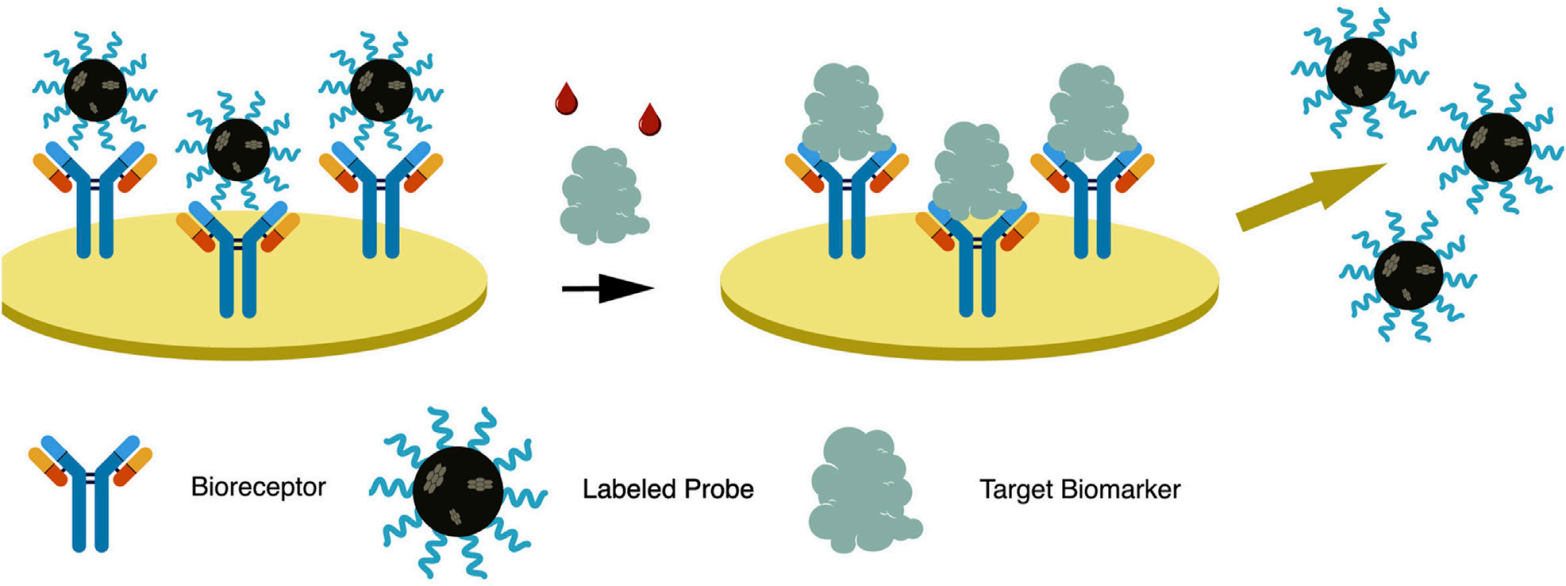
In a dual system approach—competitive format, a labeled reporter probe is released upon bioreceptor-biomarker interaction, resulting in signal generation.

The dual system approach uses a labeled secondary probe, also called the reporter probe. Two formats are usually employed utilizing this approach, the competitive and sandwich format, providing a higher sensitivity than the direct approach (Popov et al., 2021). In a competitive format illustrated in Figure 3, a labeled secondary probe is released upon the interaction of the biomarker with the primary or capture probe. In an aptasensor, methylene blue (MB) is intercalated with the complementary DNA aptamer (cDNA) sequence due to its high affinity with guanine. The aptamer and biomarker complex formation releases MB-cDNA, decreasing the redox probe’s electrochemical signal (Raouafi et al., 2019). Other nanomaterials, such as cadmium sulfide (CdS) and silver nanoparticles (AgNP), can be attached to a secondary probe, producing a stripping signal proportional to the concentration of biomarkers. Although the process is highly sensitive, its requirements of sample pre-treatment, separation, and purification of the secondary probe have limited its application (Emami et al., 2014). The redox electric signal of MB was significantly improved by using pure carbon-based electrodes, rather than the combination of graphene and gold electrodes, due to faster electron conduction velocity on the former setup (Duan et al., 2021).

**FIGURE 4:**
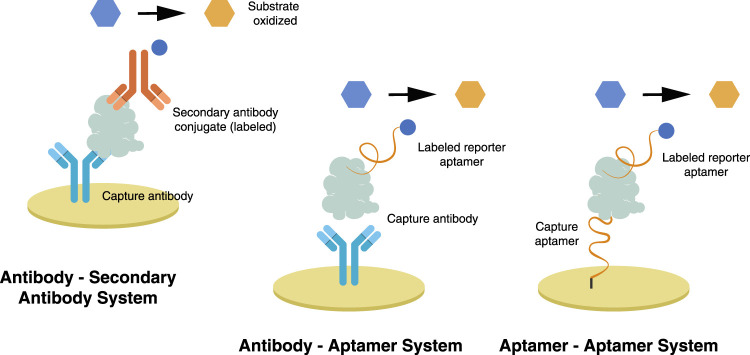
In a dual system approach—sandwich format, a capture probe is immobilized on the electrode while a labeled reporter probe attaches, thereby sandwiching the trapped biomarker.

The sandwich format in an electrochemical biosensor is a labeled method wherein an enzyme, usually HRP, is attached to a secondary probe. A primary or capture probe is used to capture the biomarker, sandwiched between the capture and enzyme-labeled secondary probe. HRP reduction catalyzes the oxidation of hydrogen peroxide, resulting in a measurable electrochemical signal (Emami et al., 2014). Among its downsides, solution-phase probe labeling may lower detection efficiency due to the diffusion limit. It may also cause contamination, especially for repetitive detections. This complicated operation of such probes is not ideal for integrated and miniaturized biosensor construction (Zhong et al., 2020). The dual system approach is illustrated in Figure 4 and is further discussed in the succeeding chapter.

## 3 Antibody, aptamer, and nanomaterial synergistic systems

The antibody, aptamer, or target antigen can be immobilized on the electrode depending on the detection approach. Proper immobilization technique will support the formation of the bioreceptor-biomarker complex on the electrode’s surface and induce signal generation. The immobilization technique must not hamper the biological activity of the bioreceptor toward the target. More importantly, it should maximize the exposure of the binding sites to the target analyte. The density of the bioreceptor should be optimized since it may hinder the binding of the target biomarker (Sharafeldin et al., 2019; Popov et al., 2021).

Different protein immobilization methods are grouped as those forming covalent bonds or non-covalent interactions with the electrode. Passive adsorption is the simplest method of protein immobilization to the electrode’s surface, using non-covalent interactions, adopted from the immobilization of antigen or antibody to ELISA microtiter plates. The main disadvantage of this method is the bioreceptors are oriented randomly on the surface, which may affect their binding capability and result in low sensitivity. Moreover, there is a massive chance of desorption during sample application and washing due to weak bonds sacrificing the reproducibility of the sensor. Bioreceptors may also undergo conformational changes, which decrease bioreactivity over time. Despite these limitations, this method is commonly used, particularly in antigen immobilization, due to its simplicity and high binding capacity (Sharafeldin et al., 2019; Popov et al., 2021). Tallapragada et al. (2017) directly immobilized antibodies on screen-printed carbon electrodes (SPCE) via passive adsorption.

Covalent linking via amine coupling is the classical and most practical technique that can be used to immobilize proteins on the electrodes’ surface. In this process, carboxyl groups must be first deposited on the surface of the electrode, which is then activated using 1:1 N-ethyl-N’-(3-(dimethylamino) propyl) carbodiimide/N-hydroxysuccinimide (EDC/NHS) (Patris et al., 2014). The electrode’s surface must develop a functional group, such as a carboxyl group (COOH), to support the linker and protein. The carboxyl group can be introduced by immersing the gold electrode or nanoparticles in an ethanol solution containing 1,6-hexanedithiol (HDT) and LiClO_4_ (Arkan et al., 2015). Mercaptopropionic acid (MPA) can also be deposited on polished gold electrodes (Chun et al., 2013), while the carbon electrode can develop COOH on its surface using sulfuric acid (H_2_SO_4_) and applying voltage (Patris et al., 2014).

Antibodies naturally possess an amine group (-NH_2_) that participates in amine coupling, while aptamers can be synthesized with amine on its 5′-end. Both bioreceptors can then be immobilized on EDC/NHS-treated surfaces, as demonstrated by Chun et al. (2013) and Nxele and Nyokong (2021). Antibodies were immobilized on rGO treated with EDC/NHS to develop a novel graphite paper-based bioelectrode (Özcan and Sezgintürk, 2022). The antigens can be immobilized in amine-functionalized rGO (Patil et al., 2022) for antibody or aptamer testing. Lysine was used to immobilize RNA aptamers into SPCE by creating a poly-L-lysine film (PLLF) layer (Bezerra et al., 2019).

Aside from the incorporation of an amine group, thiolation is commonly used to directly immobilize aptamers on gold electrodes or gold nanoparticles deposited on SPCE, as demonstrated in several studies (Salimian et al., 2017; Sattarahmady et al., 2017; Wei et al., 2018; Hassani et al., 2020). The thiol group can be easily incorporated into aptamers during their chemical synthesis. Various techniques are employed for antibodies, either taking advantage of the present functional group or incorporating a new one into the antibody. Marques et al. (2014) used SPCE nanostructured with AuNP to immobilize the capture antibody via chemisorption, while Ravalli et al. (2015) developed Au nanostructured graphite screen-printed electrode (SPE) to immobilize terminal cysteine-modified affibody. The labeled biomolecules are used while attached to AuNP as an electrochemical support. In a study, gold nanocubes were used to immobilize an HRP-labeled antibody (Shen et al., 2020).

**FIGURE 5:**
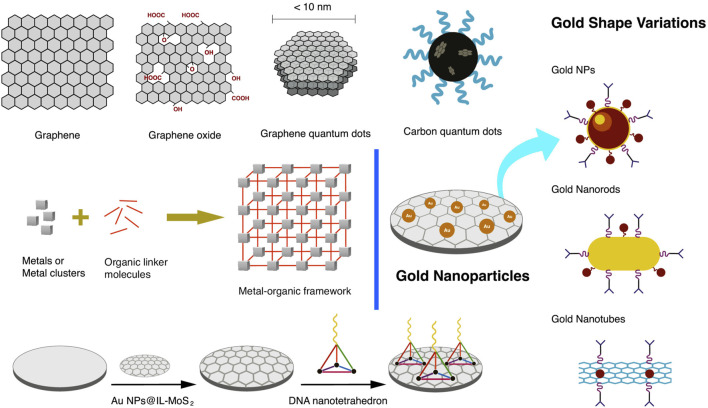
Nanomaterials are used in coating the electrode’s surface primarily to increase the surface area, anchor bio-linkers and bioreceptors, and promote electron transfer, signal amplification, and improvement of the low-end detection limit. Such nanomaterials include graphene, graphene oxide, quantum dots, metal nanoparticles, and metal-organic framework.

Incorporating AuNP into carbon-based electrodes has been a typical study in electrochemistry. AuNP has been embodied in various forms of carbon-based electrodes, from simple GCE and SPE to specially modified carbon-based electrodes. Arkan et al. (2015) used electrodeposition to grow AuNP on the surface of multi-walled carbon nanotubes in a carbon ionic liquid electrode. A paste electrode of AuNP is deposited on a multiwall carbon nanotube mixed with graphite powder. The resulting paste is tightly packed into a PVC tube with an ID of 2.0 mm, with a copper wire introduced at the other end to provide electrical contact. AuNP was used with QD, which also helped amplify faint signals from a biosensor (Johari-Ahar et al., 2015). Gold nanorods (AuNR) combined with GQD showed enhanced and new functional properties due to their cooperative interaction (Srivastava et al., 2018). AuNP shape variations are depicted in Figure 5.

Self-assembled monolayers (SAM) are formed through the spontaneous reaction of thiols with solid metal surfaces such as gold, silver, and copper. The thiolated biomolecules can arrange themselves in a well-ordered and close-packed monolayer on the gold electrode surface. Impedance measurements can be performed to characterize SAM in the absence or presence of redox species in solution (Lasia, 2014).

Random accumulation and aggregation of the bioreceptor on the electrode’s surface can impede the binding of target proteins. Immobilization techniques have been developed and improved to ensure the precise assembly and density of the biorecognition element on the electrode’s surface. Coating the electrode’s surface with nanomaterials provides support to anchor a variety of biological receptors and expand the application of the biosensor. At the same time, surface coating improves the sensitivity of the electrochemical biosensor by adding an electronic source.

AuNPs offer advantages such as biocompatibility, large efficient surface area, electrocatalytic properties, and high conductivity. Coating a glassy carbon electrode (GCE) with AuNP enhances the electron transfer rate and reduces the limit of detection. Reduced GO can also be decorated with AuNP to improve performance by reducing its tendency to aggregate upon chemical modification (Jafari et al., 2019). The catalytic activity of AuNP depends on its particle size. In the study of Wei et al. (2018), an average of 15 nm particle size was used to immobilize thiolated aptamers and improve the electrocatalytic activity provided by the electrode.

Graphene oxide (GO) is a graphene derivative with a 2D-nanostructure single atomic layered material with a significant amount of sp^3^ C-O bonds on its surface. The electrochemical reduction of GO to reduced GO includes eliminating oxygen, which boosts its electronic conductivity (Assari et al., 2019). Reduced graphene oxide (rGO) has high biocompatibility and conductivity, which is favorable for improving electrochemical signals. Moreover, rGO also introduces nanoporous structures to the electrode’s surface, resulting in a high specific surface area that can accommodate more electroactive nanomaterials and deliver a higher electrochemical response. The application of rGO in developing ultrasensitive graphite paper-based immunosensors was reported (Özcan and Sezgintürk, 2022).

Carboxyl functionalized multi-walled carbon nanotubes (MWCNT-COOH) have shown extraordinary mechanical, electrical, and thermal properties and are applied in electrochemical biosensors. The dispersion and attachment of MWCNT on the electrode are essential for more stable biomolecule attachment and electron transfer (Shamsazar et al., 2021). Incorporating iron oxide nanoparticles helps form a uniform and ordered nanocomposite layer. Due to its magnetic properties, the nanocomposite attaches tightly to the electrode (Wang et al., 2018).

Biological macromolecules can also be used as linkers to optimize the bioreceptor’s assembly. In a study by Sun et al. (2019), DNA nano-tetrahedron (NTH) anchors aptamers for its precise orientation and density. DNA NTH structure is assembled from four single-stranded nucleic acids and is firmly and homogeneously attached to the electrode’s surface. DNA NTH increases the bioreceptor’s accessibility and recognition efficiency, thereby improving the sensitivity of the electrochemical device (Sun et al., 2019).

Upon bioreceptor immobilization, surface blocking is implemented before the assay to avoid the detrimental effects of non-specific binding (Salimian et al., 2017). Common reagents for blocking include ethanolamine for EDC/NHS treated surfaces, non-fat milk, and bovine serum albumin (BSA) (Patris et al., 2014). Others employ the formation of antifouling ternary self-assembled monolayers or use antifouling polyethylene-glycol (PEG) blocking (Salimian et al., 2017).

## 4 The impact of dual system approaches to lowering the limit of detection

The electrocatalytic activity depends on the type of electrode used. Electrodes are modified with graphene oxides, metal oxides, and metal nanoparticles to increase the electrocatalytic activity while providing a platform for bioreceptor immobilization. On the other hand, the probe can be labeled to induce the redox reaction or boost the signal generation.

A single system uses one biorecognition element, an aptamer or antibody immobilized on the electrode’s surface, while the analyte is detected directly. The changes in the electrochemical measurement of a single system rely heavily on the interference caused by the immobilization of the bioreceptor and the capture of usually nonconductive biological targets. In this case, the peak current decreases in proportion to the concentration of the target analyte. Despite its setback of low peak current, most research employs this process due to its simplicity, ease of implementation, cheaper platform development, and direct measurement approach. The issue of low electronic signal generation can be circumvented by modifying the electrode or labeling the probe. For example, thionine (THI) can be attached to the electrode and used as a redox mediator to increase the electrochemical signal of an unlabeled single-system setup (Wei et al., 2018). Several studies that use a single system with labeled and unlabeled probes are presented in Table 1.

A dual system combines an antibody and a secondary antibody, two or more aptamers, and a complementary DNA aptamer, or antibodies and aptamers, in a biosensor. The main objective is to create a bio-nanocomposite that attaches to the captured target and introduces an electroactive or electrocatalytic material. A bio-nanocomposite composed of several nanomaterials often performs better than a single nanomaterial in a biosensor. The dual system is executed in two formats: the competitive format, wherein the target biomolecule displaces the secondary probe upon binding, or a sandwich format, wherein the nanocarrier of the redox catalyst attaches to the captured target. Both approaches use a capture probe immobilized on the electrode’s surface. Several articles that used a dual system approach are presented in Table 1.

### 4.1 Antibody–secondary antibody system

Sandwich-type immunoassay has been employed in most antibody-based detection, such as ELISA. This mechanism has been adopted in developing sandwich-type immunosensors, wherein the primary or capture antibody is immobilized on the electrode, and a secondary antibody attaches to and sandwiches with the target protein. The secondary antibody is tagged with an enzyme that will induce the redox reaction.

A secondary antibody tagged with HRP was used in a sandwich-type immunosensor for prostate-specific antigen (PSA) and HER2 detection. Electrocatalysis of hydrogen peroxide (H_2_O_2_) is aided by HRP, leading to higher peak current and sensitivity for PSA detection. IONP plays a vital role in redox reactions with HRP and electron exchange with the electrode (Shamsazar et al., 2021). Biniaz et al. (2017) used a biotinylated monoclonal antibody as the capture probe, while HRP and thionine-tagged polyclonal antibody was used to promote the electrocatalytic degradation of H_2_O_2_ (Biniaz et al., 2017). Other substrates used with HRP are hydroquinone/H_2_O_2_ (Patris et al., 2014) and 1,3,5-trimethylbenzene (TMB) (Tallapragada et al., 2017).

Other formats include a capture primary antibody and a biotinylated secondary antibody with high specificity to the isotype of the primary antibody. Streptavidin, a protein with a high affinity to biotin, is conjugated with HRP or alkaline phosphatase (AP), both electrocatalytic enzymes. With AP, p-nitrophenyl phosphate (pNPP) is used as a substrate that is hydrolyzed rapidly to p-nitrophenol and inorganic phosphate. AP is also used to oxidize metallic silver deposited enzymatically via 3-indoxyl phosphate (3-IP) and silver ion mixture (Marques et al., 2014; Freitas et al., 2019).

### 4.2 Aptamer–secondary/complementary aptamer

**FIGURE 6:**
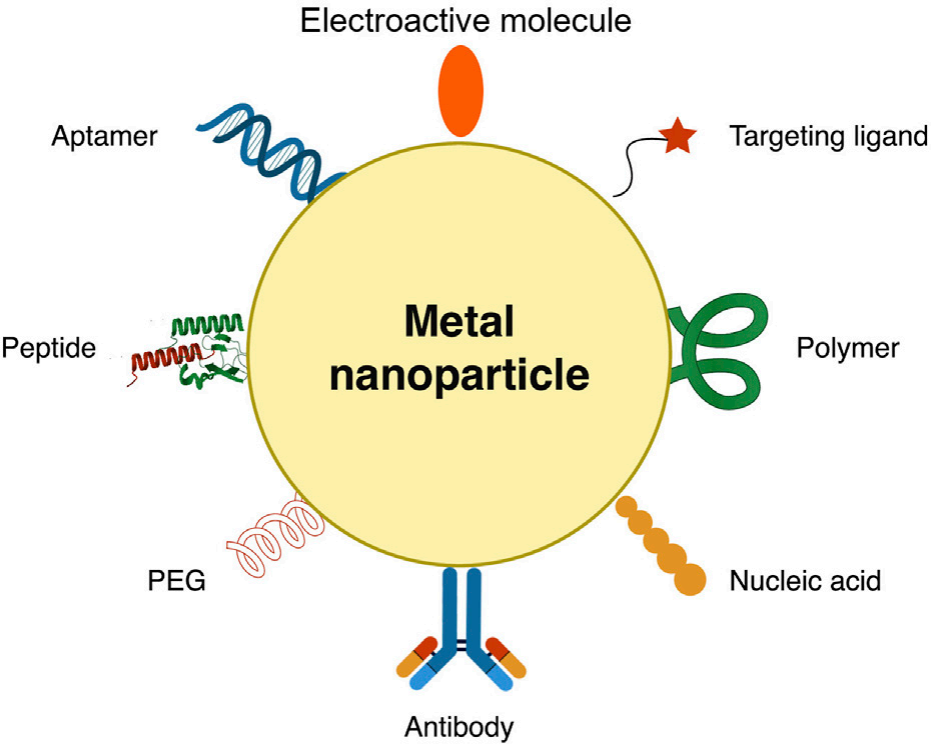
Metallic nanocarrier decorated with nanomaterials to enhance signal generation and lower limit of detection.

In a study conducted by Sun et al. (2019), Zr(IV)-based MOF known as UiO-66 was used as a shell over the IONP to form the novel magnetic MOF (MMOF IONP@UiO-66), while bimetallic nanoparticle Au@Cu was employed as a linker of the aptamer to MMOF. The capture aptamer was immobilized on a screen-printed gold electrode (SPGE) using NTH as a linker. The target is then captured by the aptamer on SPGE and sandwiched by aptamer/IONP@UiO-66. Complementary DNA attached to Au@Cu nanoparticles binds to the aptamer on IONP@UiO-66. The combination of Au@Cu and UiO-66 improves the electrocatalytic performance of IONP and amplifies the electrochemical signal while serving as nanocarriers of two types of aptamers. The increase in captured target is proportional to the amount of the nanocarriers, thus resulting in an increase in peak current by the chemical reaction: $O_{2}+2H_{2}Q\to 2Q+2H_{2}O$ and $Q+{2H}^{+}+{2e}^{-}\to H_{2}Q$ , where $H_{2}Q$ is hydroquinone, and Q is benzoquinone (Sun et al., 2019). Figure 6 depicts the nanocarrier and the common nanomaterials embedded in a metallic nanocarrier.

SPCE is coated with graphene oxide–carboxylic acid (GO-COOH) via drop-casting to immobilize aminated aptamers. A complimentary DNA (cDNA) aptamer is conjugated to the attached aminated aptamer, while methylene blue (MB) is intercalated between the aptamer and cDNA. This dual aptamer approach uses the competitive format, wherein binding the analyte will release the MB trapped between the anti-PSA aptamer-cDNA conjugate. The release of MB will increase the redox electric signal, producing a higher peak current (Raouafi et al., 2019).

### 4.3 Antibody–aptamer system

In this process, the antibody is used as a bioreceptor and an aptamer with an electrocatalytic label or incorporated in a nanocarrier (Figure 6) serves as a recognition element. Ilkhani et al. (2015) used an aptamer immobilized on magnetic beads as a capture probe for detecting the epidermal growth factor receptor (EGFR). Using magnetic beads allows an easier way of separating antigens captured by the aptamer. The antibody was conjugated to AuNP, which sandwiches the captured antigen—the increase in antigen concentration results in an increase in peak current (Ilkhani et al., 2015).

Self-assembled 2,5-*bis*(2-thienyl)-1H-pyrrole-1-(*p*-benzoic acid) or DBP and AuNP was deposited on the surface of GCE via electro-polymerization. Monoclonal anti-HER2 antibody was then immobilized to the poly-DBP@AuNP via amine coupling. In this sandwich format, the hydrazine-tagged aptamer in AuNP attaches to the captured HER2. Electrochemical measurement was performed in silver nitrate solution, wherein silver ion was reduced to silver metal by hydrazine attached to the aptamer and AuNP as a catalyst. A gradual increase in peak current was observed as the target concentration increased. As a downside, this process resulted in the deposition of reduced silver metals into the electrode (Zhu et al., 2013).

### 4.4 Comparison of LOD obtained using various detection approaches

The limit of detection (LOD) and the linear range (LR) are often used to determine the sensitivity of a biosensor. Other categories could also be examined, such as biosensor stability during implementation and storage, reproducibility, cost per analysis, and sustainability of materials used. The proposed electrochemical platform and methodology resulting in the lowest LOD based on the articles reviewed are presented in Table 2. For PSA detection, a dual aptamer system with a competitive format using MB intercalated between the capture aptamer and the complementary DNA aptamer showed the lowest LOD of 0.064 pg/mL with a dynamic LR of 0.001–100 ng/mL (Raouafi et al., 2019). Aptamers labeled with MB showed lower LOD than unlabeled aptamers, while electrochemical aptasensors performed better than immunosensors. Aptamer tagged with MB and directly immobilized on the gold electrode is the most straightforward setup, resulting in a LOD of 50 pg/mL (Sattarahmady et al., 2017).

**TABLE 2:** Cancer protein biomarker normal and infection levels and lowest reported LOD.

Biomarker	Cancer	Normal level	Infection level	Lowest reported LOD	Biosensor description
Prostate-specific antigen (PSA)	Prostate cancer	<4 ng/mL (Assari et al., 2019; Raouafi et al., 2019; Nxele and Nyokong, 2021)	4–10 ng/mL (Raouafi et al., 2019)	0.064 pg/mL (Raouafi et al., 2019)	Dual aptamer system, MB as indicator, competitive format, DPV
		<20 ng/mL (Padmavathi et al., 2017)			
Cardiac troponin I (cTnI)	Acute myocardial infarction (AMI)		5–50 ng/mL (Sun et al., 2019)	16 pg/mL (Sun et al., 2019)	Dual aptamer system, one capture and two reporter probes on nanocarrier, sandwich format, DPV
Human epidermal growth factor receptor 2 (HER2 or ErbB2)	Breast cancer	<1% positive stain (Padmavathi et al., 2017)	15–75 ng/mL (Chun et al., 2013; Bezerra et al., 2019)	0.037 pg/mL (Zhu et al., 2013)	Dual antibody-aptamer system, hydrazine label, sandwich format, SWV
		2–15 ng/mL (Bezerra et al., 2019)			
Carbohydrate antigen (CA125)	Ovarian cancer (also in lung cancer, endometrial cancer, and breast cancer)	<35 U/mL (Fan et al., 2019)		0.0016 U/mL (Johari-Ahar et al., 2015)	A single antibody on quantum dots on a gold electrode, K_3_Fe(CN)_6_ as an indicator
				0.5 pg/mL (Biswas et al., 2021)	A single antibody, ZnO nanorod, Gold electrode
Cancer antigen (CA15-3)	Metastatic breast cancer	<45 U/mL (Padmavathi et al., 2017)		0.112 U/mL (Shekari et al., 2021)	Dual aptamer system, reporter probe on nanocarrier, sandwich format, DPV
Carcinoembryonic antigen (CEA)	Breast, colorectal, and lung cancer	<5 ng/mL (cut-off range 2.5–40 ng/mL) (Padmavathi et al., 2017)	10 ng/mL	11.2 pg/mL (Shekari et al., 2021)	Dual aptamer system, reporter probe on nanocarrier, sandwich format, DPV

For cardiac troponin I (cTnI), a 16 pg/mL LOD was obtained using a multiple aptamer system, wherein one aptamer is utilized as a capture probe and two others as reporter probes, with one attached to an electrocatalytic nanocarrier. The lowest LOD (0.037 pg/mL) for HER2 detection was obtained using a dual antibody-aptamer system with a monoclonal antibody as the capture probe. The aptamer reporter probe and an electroactive label, hydrazine, were immobilized in AuNP as a nanocarrier (Zhu et al., 2013). Nanobodies applied in a dual-system immunosensor showed the highest LOD in this review, equal to 1 μg/mL (Patris et al., 2014).

In the same study, an electrochemical aptasensor performed better than an immunosensor in LOD for HER2 detection. In this case, aminated aptamers and antibodies were immobilized in separate electrodes. The results also showed the superiority of AuNP in improving the biosensor’s sensitivity (Centane and Nyokong, 2022). Srivastava et al. (2018) performed a comparative study of aptasensor vs. immunosensor using GQD and gold nanorod-modified screen-printed electrodes for PSA detection. Both setups showed comparable results with an LOD of 0.14 ng/mL (Srivastava et al., 2018). In both cases, the aptasensor outperforms the immunosensor in simplicity, cost-effectiveness, stability, and regeneration (Srivastava et al., 2018; Centane and Nyokong, 2022).

Monoclonal antibodies on cadmium selenide QD, AuNP-SiO_2_, and gold electrodes modified with MPA showed the lowest LOD of 0.0016 U/mL for CA125 detection using EIS. This ultra-sensitive electrochemical technique measures changes in electrical resistance (Johari-Ahar et al., 2015). The carbon-based electrodes with Ab on AuNP with reduced GO and AgNP with graphene QD showed a similar LOD of 0.01 u/mL measured using DPV (Fan et al., 2019; Jafari et al., 2019).

Most of the reviewed articles used DPV as an electrochemical technique. EIS showed lower LOD in a single system than DPV (Nxele and Nyokong, 2021), while SWV is more sensitive than EIS (Assari et al., 2019). Gold electrodes or the application of AuNP have exhibited lower LOD and better stability than carbon-based electrodes. Electrochemical immunosensors with sandwich format outperform single system immunosensors using an antibody as a capture element and rely on a change in resistance only. In a sandwich format, a secondary antibody labeled with biotin is used. HRP-streptavidin or ALP-streptavidin binds to biotin, which serves as an enzyme that catalyzes the substrate reduction process. This reaction produces a higher change in the electrochemical signal. Incorporation of IONP significantly decreases the LOD of the immunosensor (Shamsazar et al., 2021). A dual system using two or more aptamers, a complementary aptamer, and an antibody-aptamer system showed the lowest reported LOD for each antigen.

Table 2 shows the limit of detection of the biosensors reviewed in this article compared to the biomarkers’ average body level and infection level. Dual systems have shown detection limits lower than the infection level, indicating the high possibility of translating the research methodology into commercially viable product design.

### 4.5 Point-of-care testing adoption

Point-of-care testing (PoCT) continues to attract technology developers to produce clinically helpful PoCT devices. The critical characteristics of PoCT should match its users’ clinical and individual needs (Korte et al., 2020). Among the crucial factors described by Garg et al. (2023) that affect the translation of research output to PoCT are listed in Table 3.

**TABLE 3:** Comparison of the key characteristics of the commercially available Point-of-care test (PoCT) with the selected biosensor.

Biomarker	Brief description of technology	Limit of detection	Assay time	Sample/Test sample	Storage stability	Cost
PSA	Dual aptamer system, competitive format, DPV (Raouafi et al., 2019)	0.064 pg/mL	30 min detection time using DPV	Spiked human blood serum	stability of 98% over 2 weeks, results are reproducible over 8–10 months	Electrochemical transducer machine (one-time fee) and modified screen-printed carbon electrode (consumables)
	Lateral flow chromatographic immunoassay, semi-quantitative, cassette/strip (CTK Biotech, CA, United States)	4 ng/mL	15 min	Whole blood, serum, or plasma	1.4 months (for most immunosensors)	0.30–0.40 USD/kit (local price)
CEA	Dual aptamer system, sandwich format, DPV (Shekari et al., 2021)	11.2 pg/mL	>30 min detection time using DPV	Clinical serum samples	91% stability after 3 days of storage at 4°C, satisfactory reproducibility	Electrochemical transducer machine (one-time fee) and modified screen-printed carbon electrode (consumables)
	Immunochromatographic rapid test, quantitative, cassette (Quadratech Diagnostics, Eastbourne, United Kingdom)	5 ng/mL	5–15 min	Whole blood, serum, or plasma	Store at 4°C–30°C	0.30–0.40 USD/kit (local price)
					Shelf-life: 2 years	

The materials used in the biosensor assembly and the transduction method dramatically affect the PoCT cost. A whole gold or glassy carbon electrode is expensive and thus must be reusable to lower the testing cost. Due to this, single-use paper-based electrode modified with AuNP or other nanomaterials have gained valuable interest in PoCT due to its simplicity, low cost, stability, and ease of disposal. Electrochemical methods, such as DPV, SWV, and EIS, have been used as a readout method in PoCT. Electrochemical techniques can provide a higher sensitivity and accuracy than the usual colorimetric PoCT. However, this would require a more expensive electronic readout device that can be purchased once.

The obtained LR and LoD in all articles presented in this review are clinically relevant and highly competitive with the existing and commercially available PoCT devices. Before adoption, the proposed PoCT technology must be clinically tested. The type of sample, its volume, and collection techniques are essential considerations. Most of the current research tested the technology on spiked human blood samples.

PoCT should be accomplished within around 20 min to allow for a test and subsequent discussion of results performed within the same clinical session. Most electrochemical techniques require at least 30 min to allow ample time for sample incubation. However, this does not include the time to obtain, collect, and pre-process samples (Garg et al., 2023).

The most common PoCT is the lateral flow immunosensor, which is inexpensive and has storage stability of up to 2 years. Aptamers are more stable and affordable than antibodies and thus could improve the stability of the PoCT. However, due to the high cost of the construction materials for electrochemical biosensors, maximizing the reusability and regenerability of the product could significantly help lower its price. Table 3 presents that the electrochemical dual system aptasensor is stable at 4°C–30°C storage conditions with satisfactory reproducibility.

New opportunities in electrochemical PoCT device development have emerged in view of the developments in microfluidics, multiplexing, and machine learning. Simultaneous detection of various analytes can be achieved by using multiple aptamers in one platform. Meanwhile, integrating microfluidics into electrochemical biosensors is motivated by several perceived potential benefits such as portability, real-time monitoring, efficient sampling process, and precise detection of analyte/s, even with complex samples. This integration involves 1) the design of the microfluidic device via microfabrication techniques (e.g., soft lithography, laser ablation, or 3D printing) to include channels, chambers, and necessary features for sample introduction, mixing, flow control, delivery to the electrochemical sensor, and sample exit; 2) incorporation of electrodes onto the microfluidic chip; 3) immobilization of recognition elements onto the electrodes within the microfluidic channels; 4) provision of microfluidic structures that ensure uniform exposure of the analyte to the recognition elements on the electrochemical sensor; and 5) integrating the microfluidic-electrochemical biosensor with external systems, such as microcontrollers or data analysis software for enhanced automation, control, and data processing.

## 5 Conclusion and perspective

Incorporating nanomaterials into electrochemical biosensors has successfully improved the analytical sensitivity of the biosensor, thus allowing the detection of trace amounts of analytes relevant to clinical diagnostics. Inherent to using biological receptors are low electrical signals generated, which were overcome by labeling with ferrocene, methylene blue, and iron oxide nanoparticles or embedding the proteins in electrodes coated with metal nanoparticles, graphene oxide, and quantum dots. The bioreceptors are immobilized using organic linkers and nanoparticles with proper orientation via self-assembly, and the density can be controlled.

Aptamers pose a considerable advantage over antibodies since the former can be easily synthesized and functionalized with a label, a linker, or both. The incorporation of linkers to aptamers makes it more flexible when it comes to its immobilization and electrode development. Notable differences include the aptamer being highly efficient even as a capture element alone while using labeled complementary aptamers in a competitive or sandwich format showed the best potential.

The dual system improves the assembly of the capture probe on the surface of the electrode, regardless of whether it is carbon or gold-based. A dual system also helps increase electrocatalytic activity during measurement. The abundance of electrocatalysts on the electrode’s surface increases the peak current, thus amplifying the signal generated.

The secondary antibody, aptamer, or complementary DNA can be attached to a nanocarrier that possesses electrocatalytic properties. For the electrochemical immunosensor, the application of the secondary antibody in a sandwich format, patterned from the standard ELISA technique, significantly increased the immunosensor’s sensitivity.

The impact of using aptamers over antibodies and applying nanomaterials on the sensor’s life cycle should be examined. For developing a high-quality aptamer-based biosensor, a pragmatic approach could be using an electrochemical surface during the SELEX process. Translating the dual system electrochemical aptasensor to a PoCT device requires increasing the speed of the assay time, improving the storage stability and reusability, and performing construction materials analysis for lower assay cost.
